# Reviewed article: Santos SGP, Cruz CFR, Prezotto KH, Moreira RC, Galdino MJQ, Oliveira RR, Scholze AR, Melo EC. Temporal trend and spatial analysis of breast cancer mortality in Paraná, 2012-2021. Epidemol Serv Saude. 2025:34;e20240171

**DOI:** 10.1590/S2237-96222025v34e20240171.b

**Published:** 2025-09-08

**Authors:** Osmar de Oliveira Cardoso

**Affiliations:** 1 Universidade Federal do Piauí, Teresina, PI, Brazil

## Peer review B

## Questionnaire

Does the manuscript contain new and significant information to justify publication? Yes

Does the Abstract (Summary) clearly and accurately describe the content of the article? Yes

Is the problem significant and concisely stated? Yes

Are the methods described comprehensively? Yes

Are the interpretations and conclusions justified by the results? Yes

Is adequate reference made to other work in the field? Yes

## Manuscript Structure

Length of article is: Adequate

Number of tables is: Adequate

Number of figures is: Adequate

Please state any conflict(s) of interest that you have in relation to the review of this paper. None

Interest: Good

Quality: Excellent

Originality: Good

Overall: Excellent

Recommendation: Major Revision

## Comments

O artigo está bem elaborado e minhas preocupações com o artigo são:

1. Na figura 1 há duas linhas por macrorregião e não consigo identificar muito bem o que significa a linha preta, se são os casos, mês a mês e talvez os autores possam explicar por que há uma variação constante anual dos óbitos.
2. Seria importante os autores colocassem o total de mulheres diagnosticadas e em tratamento para comparar com o total de óbitos, verificando a taxa de letalidade da doença. Penso que isso traria mais impacto na apresentação do estudo, evidenciando ainda mais a necessidade das políticas públicas propostas na conclusão.
3. Na Figura 2 há a apresentação dos casos por município, mas o trabalho focou principalmente nas macrorregiões, então sugiro que uma figura seja feita com as macrorregiões. Se houver necessidade, como parece existir de evidenciar os municípios com maior número de casos, pode apresentar aí a imagem de georreferência apenas para essa macro com os municípios.

